# Factors Associated with Erectile Dysfunction Among Older Gay Men

**DOI:** 10.1007/s10508-024-02908-3

**Published:** 2024-06-05

**Authors:** Lucas R. Prieto, Deirdre A. Shires, Yuan Xiong

**Affiliations:** 1 Department of Social Work, College of Public Health, George Mason University, Fairfax, VA 22030 USA; 2 School of Social Work, Michigan State University, East Lansing, MI USA

**Keywords:** Erectile dysfunction, Older gay men, Sexual health, Aging, Sexual orientation

## Abstract

Erectile dysfunction (ED) is a common issue that aging men encounter, but whether internalized gay ageism (i.e., the internalization of ageist messages within the context of aging as a gay man) is related to ED among older gay men is unknown. A cross-sectional web-based survey explored the relationship between internalized gay ageism, health-related and social factors, and ED among older gay men who resided in the Midwest United States (*N* = 181). Internalized gay ageism was not significantly associated with ED. However, hierarchical regression analysis found that age (β = .224, *t* = 2.70, *p* = .008) and overall health (β = −.247, *t* = −3.05, *p* = .003) were significantly associated with ED among older gay men, suggesting that older gay men share similar risk factors for ED as the general male population. Future research should continue to explore other factors that are unique to gay men that may be associated with ED.

## Introduction

Sexual health and well-being have been shown to be associated with overall health (Davison et al., 2009; Dogan et al., 2013; Holmberg et al., 2010; Sprecher & Cate, 2004), and sex remains important to some older adults (Lindau et al., 2007). Yet, research that explores the sexual health of older gay men is lacking compared to literature that expounds on the sexual health of heterosexual and younger populations. Additionally, research is limited that examines factors associated with erectile dysfunction (ED; a common health condition among aging men; Laumann et al., 2007), which is linked with negative health outcomes, such as an increased risk of depression (Liu et al., 2018).

Erectile function is a critical mechanism for many cisgender men to express their sexuality and engage in sexual activities. The “inability to achieve or maintain an erection,” known as ED (Morgentaler, 1999), often threatens the ability of men to engage in certain sexual activities or sexual behaviors, such as penetration (Smith et al., 2007). It has been estimated that over 20% of men aged 40 or older have experienced some form of ED, and this percentage increases with age (Laumann et al., 2007). Complications from ED are known to decrease overall sexual satisfaction (Smith et al., 2007). ED may also cause stress, anxiety, depression, or low self-confidence (Latini et al., 2006; Shabsigh et al., 1998; Tomlinson & Wright, 2004).

### Health-Related Factors and Erectile Dysfunction

Prior studies have suggested that ED may be caused by several physical and psychological factors. For instance, literature suggests that one independent risk factor for ED diagnosis or treatment is age (Mulhall et al., 2016). Erectile function has been linked to cardiovascular health (Gandaglia et al., 2014), diabetes (Penson et al., 2009), and prostate cancer (and treatment; Nelson et al., 2011). Additionally, normal blood flow to the penis is essential for erectile function. Hypertension or high blood pressure may prevent dilation of the penis (i.e., erection) because blood may be trapped, preventing it from entering the penis (Burchardt et al., 2000; Feldman et al., 1994; Foy et al., 2019; Heikkilä et al., 2017). A meta-analysis found that ED occurs 1.39 times more in depressed patients than patients without depression (Liu et al., 2018), and another study found a bidirectional relationship between the two (Shiri et al., 2007). Behavioral and mutable factors such as cigarette smoking (Mannino et al., 1994; Tostes et al., 2008), lack of regular exercise (Silva et al., 2017), side effects from taking medication (Rosen & Marin, 2003), and cannabis use (Pizzol et al., 2019) are also associated with ED.

ED is more common among gay men than heterosexual men by 1.5 times (Bancroft et al., 2005; Barbonetti et al., 2019), yet little is known about how these factors play a role in ED among older gay men. Additionally, several of these physical and mental health factors that are associated with ED in the general population are more prevalent among gay men (Gonzales & Henning-Smith, 2017; Operario et al., 2015). For instance, hypertension (Jackson et al., 2016) and illicit drug use are more common (Rhodes, et al., 2007) in gay men compared to heterosexual men. Thus, gay men may be at increased risk of experiencing ED.

### Social Factors and Erectile Dysfunction

There is evidence suggesting that factors associated with ED go beyond health factors (Aytaç et al., 2000). For instance, studies have shown that some racially and ethnically diverse men are at a heightened risk of ED (Saigal et al., 2006; Selvin et al., 2007; Smith et al., 2009). Thus, it is essential to examine the potential influence of social factors on ED among older gay men. For example, internalized gay ageism, a term coined by Wight et al. (2015), is the internalization of ageist messages in the context of aging as a gay man, and may contribute to ED. Understanding social factors is particularly important given the vast differences between gay and straight cultures. Gay culture reinforces ageist stereotypes (Slevin & Linneman, 2010), and said stereotypes may lead to feelings of increased self-consciousness about their bodies and reduced sexual self-esteem among gay men in general (Filice et al., 2019; Slevin & Linneman, 2010). These permissible acts of ageism in the gay community may stem from the internalized homophobia endured by gay men and the effects of the HIV/AIDs crisis (Meyer, 1995). Therefore, such factors could potentially play a role in ED among older gay men, especially as there is emerging evidence that suggests a negative relationship between internalized ageism and sexual health and well-being among older adults in the general population (Syme & Cohn, 2021). However, little is known about the influence of internalized gay ageism on ED among aging gay men.

To guide this study, we adapted the stereotype embodiment theory (Levy, 2009), which posits that individuals unknowingly internalize stereotypes, influencing health outcomes through pathways, such as a physiological pathway. We hypothesized that older gay men who report higher rates of internalized gay ageism will report higher rates of ED, and this relationship will be mediated by blood pressure, even when sociodemographic characteristics and other health-related factors known to influence ED are controlled.

## Method

### Participants

The Study on Aging and Sexual Satisfaction Among Gay Men was a cross-sectional online survey that assessed sexual health and well-being among gay men 50 years or older who resided in the Midwestern United States. Eligibility for participation in the survey included being 50 years or older (assessed by “What is your age range?”), (2) identifying as gay (assessed by “Do you identify as gay?”), (3) having been assigned male at birth (assessed by “What sex was originally listed on your birth certificate?”), (4) identifying as male (assessed by “What is your primary gender identity today?”), and (5) residing in a Midwestern state (Illinois, Indiana, Iowa, Kansas, Michigan, Minnesota, Missouri, Nebraska, North Dakota, Ohio, South Dakota, and Wisconsin) at the time of the survey.

Data were collected from December 2021 to May 2022 using Qualtrics online survey software (a paper survey option was available upon request). The survey took participants approximately 15 to 20 min to complete. Recruitment of the sample was accomplished through several avenues, including word of mouth, email blasts, phone calls, and presentations. Places of recruitment included a combined total of over 300 contacts to lesbian, gay, bisexual, and transgender + organizations, word-of-mouth contacts, and several personal contacts among others. A total of 195 responses were collected and after data cleaning, 181 participants were analyzed. Informed consent language was displayed at the beginning and end of the survey. A $10 Amazon gift card incentive was offered to participants who completed the survey and provided their home mailing address on a separate form linked to the main survey.

## Measures

### Dependent Variable

*Erectile Dysfunction* ED was measured by using investigator-adapted items from a subscale of the Gay Male Sexual Difficulties Scale (McDonagh et al., 2016). The subscale used four items on a 6-point scale: *0*-*not applicable* to *5*-*all the time*. The original question asked, “During the past 6 months…” and was adapted to ask “During the past 12 months….” The four items for the scale included (1) “When you engaged in sexual activity, were you able to get an erection?” (2) “When you wanked (i.e., jerked), were you able to get an erection?” (3) “When you engaged in sexual activity, were you able to maintain your erection (i.e., keep it up)?” and (4) “When you wanked, were you able to maintain your erection?” Two of the response options were adapted to say “jerked off” instead of “wanked” to reflect American slang for masturbation. The scale score for ED was computed based on the mean score of the four items (non-applicable responses did not count toward the score and were recoded as “missing”) from the ED subscale of the Gay Male Sexual Difficulties Scale (McDonagh et al., 2016). The theoretical and actual range of scores was 1–5, with higher scores indicating higher ED. The final score was set to “missing” if there were any missing values for the four items. Cronbach’s alpha was (α = 0.94) with a skewness of 0.10.

### Independent Variables

*Internalized gay ageism* Internalized gay ageism was measured using Wight et al.’s (2015) Internalized Gay Ageism Scale, which consisted of six statements. These six statements were: “As I get older, I feel good about myself as a gay man”; “I feel that older gay men are respected in the gay community”; “Aging is especially hard for me because I am a gay man”; “I am not too worried about looking older”; “As I get older, I feel more invisible when I am with other gay men”; and “I feel pressured to try to look younger than my age.” The response options (utilizing Wight et al. (2015) Table 1 response categories) ranged from 1 (strongly disagree) to 4 (strongly agree). The questions “As I get older, I feel good about myself as a gay man,” “I am not too worried about looking older,” and “I feel pressured to try to look younger than my age” were reverse coded. The scale score for internalized gay ageism was computed by taking the mean of the six items from the Internalized Gay Ageism Scale (Wight et al., 2015). The final score was set to “missing” if there were any missing values for any of the six items. Cronbach’s alpha was (α = 0.77) with a skewness of − 0.23. Higher scores indicated higher internalized gay ageism.

**Table 1:** Descriptive characteristics of Midwestern gay men 50 years or older (N = 181)

Variable		*n*		%
*Sociodemographic characteristics*				
Age (in years) (M/SD)		176		65.29, 9.32
Missing		5		2.8
Education				
Some college or technical school or below		35		19.3
Undergraduate degree		63		34.8
Graduate degree		83		45.9
Missing		0		0
Race/ethnicity				
Black		15		8.3
White		155		85.6
Other/multiracial and multiethnic		11		6.1
Missing		0		0
Income				
Less than $25,000		18		10.2
$25,000 to less than $35,000		20		11.3
$35,000 to less than $50,000		26		14.7
$50,000 to less than $75,000		27		15.3
$75,000 to or more		86		48.6
Missing		4		2.2
Relationship type				
Single or widowed		91		50.3
Monogamous		34		18.8
Monogamish		26		14.4
Open		29		16.0
Missing		1		.6
Residence				
Urban		87		48.1
Suburban		63		34.8
Small city		21		11.6
Rural		9		5.0
Missing		1		.6
*Health status*				
HIV status				
Positive		33		18.2
Negative		147		81.2
Missing		1		.6
Overall health scale^a^		179		14.8, 3.58
Missing		2		1.1
High blood pressure				
Yes		91		50.3
No		90		49.7
Missing		0		0
Depression				
Depression (clinically diagnosed or reported symptoms)		86		47.5
Never been diagnosed or showing symptoms of depression		91		50.3
Missing		4		2.2
Diabetes				
Yes		35		19.3
No		146		80.7
Missing		0		0
Prostate cancer				
Yes		15		8.3
No		166		91.7
Missing		0		0
Erectile dysfunction^b^		151		2.23, 1.5
Missing		30		16.6
*Health behaviors*				
Physical activity level				
Not at all		17		9.4
Less than once a week		29		16.0
1–2 times a week		37		20.4
3 times a week		38		21.0
More than three times a week but not every day		42		23.2
Every day		17		9.4
Missing		1		.6
Smoking status				
Current		21		11.6
Former		73		40.3
Never		84		46.4
Missing		3		1.7
PDE5 inhibitor use				
Yes		60		33.1
No		121		66.9
Missing		0		0
Alcohol use before/during sex				
Yes		62		34.3
No		117		64.6
Missing		2		1.1
Illicit drug use before/during sex				
Yes		46		25.4
No		135		74.6
Missing		0		0
Social factors				
Internalized gay ageism^c^		181		2.21, .55
Missing		0		0
Experienced ageism^d^		181		1.06, 1.49
Missing		0		0
Internalized homophobia^e^		177		1.41, .61
Missing		4		2.2
Comfortable with health provider				
Very comfortable		96		53.0
Somewhat comfortable		57		31.5
Not comfortable		27		14.9
Missing		1		.6

*M* Mean; *SD* Standard Deviation

^a^Overall health scale was computed by summing four items from the Self-Rated Health Scale (Turner et al., 2016). Responses ranged from definitely true to definitely false, and scores were summed across the four items and higher scores indicated better health. A theoretical range of 4–20 and an actual range of 6–20. Cronbach’s alpha was (α = .71) with a skewness of −.25

^b^Erectile dysfunction was measured by using a subscale from the Gay Male Sexual Difficulties Scale (McDonagh et al., 2016). The subscale used four items on a 6-point scale from not applicable to all the time. The four items were reverse scored in the original scale and in the current study resulted in higher scores indicating more erectile difficulties (α = .94) with a skewness of .10. The mean was computed based on the number of items a participant answered (non applicable responses did not count toward their score and was recoded as missing). Theoretical range of 1–5 and actual range of 1–5

^c^Internalized gay ageism was assessed from the Internalized Gay Ageism Scale (Wight et al., 2015) using six items on a 4-point scale ranging from *strongly disagree* to *strongly agree*. Three items were reverse coded. The scale was computed by averaging each item (α = .77), and the scale had a skewness of -.23. Higher scores indicated higher levels of internalized gay ageism. Theoretical range of 1–4 and actual range of 1–4

^d^Experienced ageism was assessed from the created Ageism Scale (Wight et al., 2015). Experienced ageism scoring was a count of seven items with scores ranging from 0 to 7 (actual range 0–7; α = .73, skewness 1.65). Higher scores indicated more experienced ageism

^f^Internalized homophobia was computed by summing the responses to each item and dividing by the total number of items (Herek et al., 2009). The theoretical scale score was 1–5 and actual score range was 1–4.40 with a skewness of 1.88 (α = .80). Higher scores indicated more experienced ageism

*Sociodemographic variables* Participant demographic items included age, education level, race/ethnicity, relationship type, income, and residence.

*Age* Age was ascertained with the open-text question, “What is your age in years (whole numbers)?”

*Education* Education level was assessed based on the 2019 Behavioral Risk Factor Surveillance System survey (Centers for Disease Control & Prevention, 2019). The question was, “What is the highest grade or year of school you completed?” Response options were adapted, and they included less than high school, some high school, some college or technical school, community college degree (e.g., A.A.), undergraduate degree (e.g., B.S., B.A. etc.), graduate degree (e.g., M.S.W., M.A., Ph.D., J.D., M.D. etc.). Responses were collapsed into three categories: community college or below, undergraduate degree, and graduate degree.

*Race and ethnicity* To ask about race and ethnicity, a question from Hughes et al. (2016a) “Rethinking and Updating Demographic Questions: Guidance to Improve Descriptions of Research Samples” was used. Participants were asked, “Which categories describe you? Select all that apply to you” with response options of “American Indian or Alaska Native”; “Asian”; “Black or African American”; “Hispanic, Latino or Spanish origin”; “Middle Eastern or North African”; “Native Hawaiian or other Pacific Islander”; “White”; and “Some other race, ethnicity, or origin (please specify).” The analytic variable used three categories: Black, White, and other/multiracial and multiethnic (e.g., Hispanic, Latino, or Spanish: Middle Eastern or North African, Native Hawaiian or other Pacific Islander, and Some other race, ethnicity, or origin [please specify].

*Income* Income was assessed using an adapted question from the 2019 Behavioral Risk Factor Surveillance System (Centers for Disease Control & Prevention, 2019). It asked, “What is your annual household income from all sources?” The response options were adapted to include “don’t know/not sure,” “less than $25,000,” “$25,000 to less than $35,000,” “$35,000 to less than $50,000,” “$50,000 to less than $75,000,” and “$75,000 or more.” Don’t know/not sure was considered missing.

*Relationship type* Relationship type was assessed using an investigator-adapted version from Parsons et al. (2013). It asked, “What best describes your current relationship type?” The response options were “single (e.g., do not have main partner),” “monogamous (e.g., have a partner and agreed to only have sex with each other and no sex with casual partners),” “monogamish (e.g., have partners and agreed to have sex with others but only when the other member of the relationship was present),” “open (e.g., have a partner and both the partner and I have casual partners without the other partner present),” and “other (please specify in the box).” Single” and “widowed” were combined into one category for analysis.

*Residence* This question was adapted from the Michigan Transgender Health Survey 2018 (Kattari et al., 2020) and asked: “Would you consider where you live to be…?” Our response options included: “urban (metropolitan areas; cities of over 100,000 people [e.g., Detroit, Cleveland, Chicago, Milwaukee]),” “suburban (neighborhoods on the outskirts of near larger cities,” “small city (cities of 10,000 to 100,000 people [e.g., Jackson, Port Huron, Saginaw]), and “rural (villages, hamlets, towns, cities under 10,000 people).”

### Social Variables

*Experienced ageism* Experienced ageism was measured using an investigator-adapted version of Wight et al.’s (2015) Ageism scale. The adaptation to Wight et al. (2015) scale changed the wording to ask about the past 12 months. The scale assessed if participants had any occurrence within the past 12 months of the following acts or impressions attributed to one’s age: “bullied,” “made fun of by a stranger/strangers,” “ignored by others,” “called a derogatory name,” “rejected by younger people,” “not taken seriously,” and “treated like a child” with “yes” or “no” responses. The scale score for experienced ageism was taking the sum score of seven items from the Ageism Scale (Wight et al., 2015). Answers of “yes” were given a score of 1 and answers of “no” a score of 0. The actual range was a minimum score of 0 and a maximum score of 7, with higher scores indicating more experienced ageism. Cronbach’s alpha was (α = 0.73) with a skewness of 1.65.

*Internalized homophobia* The scale used to measure internalized homophobia was the Revised Internalized Homophobia Scale (Herek et al., 2009). Example statements include, “I wish I weren’t gay” and “If someone offered me the chance to be completely heterosexual, I would accept the chance.” Response options were on a 5-point Likert scale and ranged from 1 (*disagree strongly*) to 5 (*agree strongly*). The computed scale score took the sum of the five items (Herek et al., 2009) and divided by the total number of items, with higher scores indicating more internalized homophobia. The final score was set to “missing” if there were any missing values for the five items. The computed scale had a Cronbach’s alpha of (α = 0.80) and skewness of 1.88. The theoretical score range was 1–5 and actual score range was 1–4.40.

*Comfortable with health provider* Comfortability with health provider was measured by asking “How comfortable are you discussing your sexual health and well-being with your health provider?” Responses included “not comfortable”, “somewhat comfortable,” and “very comfortable.”

### Health Status Variables

*HIV status* HIV status was measured by asking participants, “Have you ever been told by a health care provider that you had HIV and/or AIDS?” with “yes” or “no” response options. These response options were investigator adapted from “either or both diagnoses” and “none” from a study that explored HIV disparities among older gay and bisexual men (Emlet et al., 2020).

*Overall health* Overall health was measured using the Self-Rated Health Measure (Turner et al., 2016). Participants were asked how much they agreed with four statements such as, “You seem to get sick a little easier than other people” and “In general, your health is excellent.” Responses ranged from 1 (*definitely true*) to 5 (*definitely false*). Overall health was computed by summing the four items, with higher scores indicating better health. The scale was adapted to ask respondents to indicate their agreement within the last 12 months. The final score was set to “missing” if there were any missing values for the four items. The scale had a theoretical range of 4–20 and an actual range of 6–20. Cronbach’s alpha was (α = 0.71) with a skewness of −0.25.

*High blood pressure* A 2011 Behavioral Risk Factor Surveillance System (Centers for Disease Control & Prevention, 2011) question was used to measure high blood pressure. It asked, “Have you EVER been told by a doctor, nurse, or other health professional that you have high blood pressure?” The response options for this question consisted of “yes,” “no,” “told borderline high or pre-hypertensive,” and “don’t know/not sure.” The high blood pressure analytic variable was collapsed into a binary of “yes” or “no” with “borderline high or pre-hypertensive” and “don’t know/not sure” coded as “no.”

*Depression* Depression was measured using the items from the Patient Health Questionnaire-2 scale (Kroenke et al., 2003) and an adapted depression question from the 2011 Behavioral Risk Factor Surveillance System (BRFSS; Centers for Disease Control & Prevention, 2011). The BRFSS question was, “Has a doctor, nurse, or other professional ever told you that you had a depressive disorder (including depression, major depression, dysthymia, or minor depression)?” with adapted response options of “yes,” “no,” or “don’t know/not sure” (“refused” was removed as a response option). Participants who scored a 3 or above on the PHQ-2 scale or answered “yes” to the BRFSS question were considered clinically diagnosed with depression or had depression symptoms. Those who answered “no” and scored less than 3 on the PHQ-2 scale were considered not to be clinically diagnosed or have reported symptoms.

*Diabetes* Diabetes was measured using an adapted question from the 2005–2006 CDC Diabetes Questionnaire (National Health and Nutrition Examination Survey, 2007). Participants were asked, “Have you ever been told by a doctor or other health professional that you have diabetes?” with answers of “yes,” “no,” “pre-diabetes or borderline diabetes,” “no,” and “don’t know/not sure.” The words “sugar diabetes” were removed from the question stem. Diabetes was collapsed into a binary variable of “yes” or “no” with “pre-diabetes or borderline diabetes” “no,” and “don’t know/not sure” coded as “no.”

*Prostate cancer* Prostate cancer was measured by asking, “Has a doctor ever told you that you had prostate cancer?” with responses of “yes,” “no,” and “don’t know/not sure.” A binary variable was created with “yes” and “no” categories and “don’t know/not sure” was considered “no.” This question was adapted from 2019 Behavioral Risk Factor Surveillance Survey questions about other diseases (Centers for Disease Control & Prevention, 2019).

### Health Behavior Variables

*Physical activity level* To obtain information about a participant’s level of physical activity, a question from the Physical Activity Measure was used (Brown, 2011). The question asked, “In general how often do you participate in moderate or intense physical activity for at least 30 min? Moderate physical activity will cause a slight increase in breathing and heart rate such as brisk walking.” Response options were “not at all,” “less than once a week,” “1–2 times a week,” “3 times a week,” “more than three times a week but not every day,” and “every day” with higher scores indicating more physical activity.

*Smoking status* Two questions from the 2019 BRFSS (Centers for Disease Control & Prevention, 2019) were used to assess smoking status. The first question asked, “Have you smoked at least 100 cigarettes in your life?” with responses of “yes” and “no.” Response options refused and don’t know/not sure were removed. Those who selected “yes” were asked, “Do you smoke cigarettes every day, some days, or not at all?” Response options included “every day,” “some days,” “not at all,” and “don’t know/not sure.” The final analytic variable combined responses from both questions to create three categories of smoking status: current, former, and never. Those who answered “yes” to the first question and “not at all” or “don’t know/not sure” were coded as former smokers. Participants who answered “yes” to the first question and “some days” or “every day” to the second question were considered current smokers. Participants who answered “no” to the first question and “not at all” or “don’t know/not sure” to the second question were considered never smokers.

*Phosphodiesterase inhibitor use* Phosphodiesterase-5 (PDE5) inhibitor use was measured by asking participants, “In the past 12 months, have you used PDE5 inhibitors (such as Viagra or Cialis) for sexual encounters?” with “yes” or “no” response options.

*Alcohol use before or during sex* Alcohol use during sex was measured using one question from the Substance Use Measure (Knyazev et al., 2004), which was adapted to ask about alcohol use immediately before or during sex. The original question was “Have you used alcohol?” The revised question was, “In the past 12 months, have you used alcohol immediately before or during sex?” Response options were “yes” or “no.”

*Illicit drug use before or during sex* Illicit drug use before or during sex was measured by using an adapted question item that asked participants about drug use in general (Knyazev et al., 2004). The original question stated, “Have you ever tried drugs?” The adapted question was, “In the past 12 months, have you used illicit drugs immediately before or during sex (e.g., marijuana, ketamine, poppers, crystal meth, heroin, etc.)?” with adapted response options of “yes” or “no.”

### Statistical Analysis

All statistical analyses were conducted using IBM SPSS V.28 (IBM SPSS Statistics for Windows, 2021). Descriptive analyses were conducted to summarize all variables of interest. Means and standard deviations were measured for continuous variables, and frequencies and percentages were calculated for categorical variables. Bivariate analyses were conducted using non-parametric Mann–Whitney tests to test the relationship between categorical independent variables with two groups and the outcome variable of ED, and Kruskal–Wallis tests were conducted to test the relationship between categorical independent variables with three or more groups and ED. For continuous variables that were non-normally distributed, Spearman’s r test was conducted.

A mediation analysis was conducted between internalized gay ageism and ED with a potential mediator of blood pressure. However, at the bivariate level internalized gay ageism and ED were not significantly associated; therefore, a hierarchical linear regression was chosen. A four-stage hierarchical linear regression was conducted to explore the relationship between predictor variables that were significant *p* < .10 level in the bivariate analysis and ED. To ensure sufficient power to detect significant differences in the outcome variable we previously suggested guidelines for regression modeling (Harrell, 2001). An alpha of 0.10 for the bivariate analysis was selected to limit the number of variables in the multivariate model. Internalized gay ageism, although not significant, was included for theoretical reasons. In the first model, sociodemographic characteristics were entered (i.e., age, race and ethnicity, and residence). In the second model, health variables were entered (i.e., overall health, HIV status, and blood pressure). In the third model, health behavior variables were entered (i.e., physical activity, alcohol use before and during sex). In the final model, internalized gay ageism was entered.

## Results

### Participant Characteristics

Among the 181 participants, the mean age was 65.29 years (SD = 9.32). The majority had an undergraduate degree (34.8%) or graduate degree (45.9%) degree and were White (85.6%). Slightly over half of participants were single or widowed compared to being in some form of a relationship (50.3% versus 49.2%). Nearly half of participants resided in an urban setting (urban 48.1% versus suburban 34.8%, small city 11.6%, and rural 5.0%). Most participants were HIV negative (81.2%) and a little more than half indicated they had high blood pressure (50.3%). About half had been clinically diagnosed or indicated symptoms of depression (47.5%). The mean average of rated overall health was 14.8 (*SD* = 3.58) and a majority of participants reported that they exercised at least one or two times a week or more. The mean internalized gay ageism score was 2.21 (*SD* = .55), the mean experienced ageism score was 1.06 (*SD* = 1.49), and mean internalized homophobia score was 1.41 (*SD* = .61). Most did not use PDE5 inhibitors 66.9%, illicit drugs 74.6%, or alcohol 64.6% before/during sex. The mean ED score was just over 2 (*M* = 2.23, *SD* = 1.5) meaning on average participants reported having ED “once or twice” in the past 12 months (see Table 1).

### Bivariate Analysis

In bivariate analyses, the variables that were associated with ED at the *p* < .10 were alcohol use before/during sex (*p* = .052), physical activity level (*p* = .039), and race/ethnicity (*p* = .035; see Table 2), HIV status (*p* = .084) and high blood pressure (*p* = .098; Table 2).

**Table 2:** Bivariate analyses of Midwestern gay men 50 years or older and erectile dysfunction (N = 181)

Variable		*n*	Mean rank	U/KW	*p*
*Sociodemographic characteristics*					
Education	Community college or below	27	82.43	.79	.678
	Undergraduate degree	52	73.51		
	Graduate degree	72	75.39		
	Total	151			
Race/ethnicity	Black	12	74.96	6.71	.035
	White	129	78.74		
	Other/multiracial and multiethnic	10	41.85		
	Total	151			
Income	Less than 25,000	15	83.97	1.80	.773
	25,000 to less than 35,000	13	81.77		
	35,000 to less than 50,000	23	74.22		
	50,000 to less than 75,000	20	66.95		
	75,000 or more	77	73.47		
	Total	148			
Relationship type	Single/widowed	66	79.60	2.52	.470
	Monogamous	31	69.76		
	Monogamish	25	80.88		
	Open	28	67.39		
	Total	150			
Residence	Urban	69	69.25	6.25	.100
	Suburban	57	82.02		
	Small city	17	88.41		
	Rural	7	52.64		
	Total	150			
*Health variables*					
HIV Status	Positive	28	88.18	1353.00	.084
	Negative	122	72.59		
	Total	150			
Blood pressure	Yes	75	81.88	2409.00	.098
	No	76	70.20		
	Total	151			
Depression	Clinical diagnosis or showing signs of depression	66	79.08	2469.50	.299
	No clinical diagnosis or signs of depression	83	71.75		
	Total	149			
Diabetes	Yes	27	86.89	1380.00	.150
	No	124	73.63		
	Total	151			
Prostate cancer	Yes	12	70.42	767.00	.642
	No	139	76.48		
	Total	151			
*Health behaviors*					
Physical activity	Not at all	14	92.54	11.68	.039
	Less than once a week	22	93.61		
	1–2 times a week	30	79.52		
	3 times a week	32	62.69		
	More than three times a week but not every day	35	73.50		
	Every day	17	59.18		
	Total	150			
Smoking status	Current	19	65.16	1.48	.477
	Former	53	78.81		
	Never	76	73.83		
	Total	148			
PDE5 inhibitors	Yes	55	82.59	2277.50	.158
	No	96	72.22		
	Total	151			
Drug use before/during sex	Yes	42	69.25	2005.50	.235
	No	109	78.60		
	Total	151			
Alcohol use before/during sex	Yes	56	66.21	2112.00	.052
	No	93	80.29		
	Total	149			
*Social variables*					
Comfort with health provider	Very comfortable	85	70.11	3.64	.162
	Somewhat comfortable	45	84.47		
	Not comfortable	21	81.69		
	Total	151			
		*r*		*P*	
Age		.28		< .001	
Overall health		-.35		< .001	
Internalized gay ageism		-.02		.811	
Experienced ageism		.02		.825	
Internalized homophobia		.03		.753	

*U* Mann–Whitney U Test, *KW* Kruskal–Wallis Test

Age was positively associated with ED (*r* = 0.28, *p* < .001), while overall health was negatively associated with ED (*r* = −.35, *p* < .001). Notably, internalized gay ageism (*p* = .811) and experienced ageism (*p* = .825) were not significantly associated with ED.

### Hierarchical Linear Regression Analysis

#### Model 1

In Model 1, age positively predicted ED, β = 0.287, *t* = 3.43,* p* < .001. Age, race and ethnicity, and residence accounted for 10.2% variation in ED and contributed significantly to the model *F*(6, 134) = 3.66, *p* = 0.002 (data not shown).

#### Model 2

In Model 2, age remained significant, β = 0.264, *t* = 3.26, *p* = .001. Overall health negatively predicted ED, β = −0.286, *t* = −3.69, *p* < .001 (see Table 3). Introducing overall health, HIV status, and blood pressure explained an additional 19.3% of variation in ED and significantly contributed to the model *F(*3, 131) = 6.05, *p* < .001 (data not shown).

**Table 3:** Hierarchical regression analysis of predictors of erectile dysfunction

Predictor variables	Regression 1	Regression 2	Regression 3	Regression 4
*Sociodemographic variables*				
Age	.287***	.264**	.246**	.224**
Race and ethnicity				
Black (Reference)				
White (1, yes; 0, no)	− .066	− .075	− .046	− .031
Other/multiracial and multiethnic (1, yes; 0, no)	− .226*	− .218*	− .199	− .201
Residence				
Urban (reference)				
Suburban (1, yes; 0, no)	− .024	− .028	− .034	− .030
Small city (1, yes; 0, no)	.084	.093	.074	.082
Rural (1, yes; 0, no)	− .121	− .094	− .086	− .101
*Health variables*				
Overall health		− .286***	− .231**	− .247**
HIV status (1, positive; 0, negative)		.078	.081	.085
High blood pressure (1, yes; 0, no)		.116	.099	.099
*Health behavior variables*				
Physical activity (1, not at all; 6, every day)			− .141	− .142
Alcohol use before and during sex (1, yes; 0, no)			− .114	− .116
*Social variables*				
Internalized gay ageism				− .3089
*R*^*2*^	.141	.245	.279	.286
Adjusted R square	.102	.193	.218	.219
*R*^*2*^ change	.141	.105	.034	.007

Standardized coefficients betas, *p < 0.05; **p < 0.01; ***p < 0.001

#### Model 3

In Model 3, age remained significant, β = 0.246, *t* = 3.05, *p* = .003. Overall health remained significant in Model 3, β = −0.231, *t* = −2.90, *p* = .004 (see Table 3). Adding physical activity and alcohol use before and during sex explained an additional 21.8% of the variance in ED and significantly contributed to the model *F*(2, 129) = 3.03, *p* = .052 (data not shown).

#### Model 4

In Model 4, age (β = 0.224, *t* = 2.70, *p* = .008) and overall health (β = −0.247, *t* = −3.05, *p* = .003) remained significant. Neither physical activity nor alcohol use before and during sex remained significant in Model 4. Finally, adding internalized gay ageism to the final model explained 0.07% of variance of ED and was insignificant to the model *F*(1, 128) = 1.25, *p* = .265 (data not shown). In terms of effect size, R^2^ for Model 4 is 0.286, and adjusted R^2^ was 21.9%.

## Discussion

This study helps to fill in the gap in knowledge about the sexual health of older gay men by examining a wide range of established and unique potential predictors of ED among a sample of gay men aged 50 years or older in the Midwest. Among this sample, participants reported experiencing ED once or twice in the past 12 months on average, and before controlling for other factors, ED was associated with age, overall health, physical activity, and alcohol use before and during sex. In line with previous research among the general population of men (Laumann et al., 2007), we found that increasing age and decreasing overall health were both predictors of ED.

Contrary to our hypothesis, internalized gay ageism was not a significant predictor of ED in this sample. There are explanations for this null finding. First, participants reported low levels of internalized gay ageism on average. In addition, our study consisted of individuals who were highly educated, mostly White, and of high socioeconomic status, and who therefore may have access to health or mental health services and other advantages that could lead to decreased experiences of both internalized gay ageism and ED (Williams & Cooper, 2019), potentially influencing a reduction in both internalized gay ageism and ED. Thus, future studies may benefit from exploring the association between internalized gay ageism and ED with a larger and more diverse sample of older gay men. Lastly, a recent study found that self-directed ageism was associated with sexual functioning among older adults (Gitliz & Ayalon, 2023). Although a nonsignificant result was found in our study for similar variables, the effect sizes were in the same direction and similar magnitude; self-directed ageism and sexual function (β = −0.18, *p* = .02) (Gitliz & Ayalon, 2023) and internalized gay ageism and ED (β = −0.089, *p* = .265). Therefore, it is possible that our study was underpowered. Alternatively, older sexual minorities may be better prepared to handle bias. For example, a previous pilot study found that older sexual minorities reported having lower perceived ageism compared to older straight adults, potentially due to resilience factors associated with being a sexual minority (Flesia et al., 2023). Therefore, our finding of low internalized gay ageism and the lack of association between internalized gay ageism and erectile function could be attributed to an explanation similar to the one given by Flesia et al. (2023) for perceived ageism.

Alcohol use is known to be more prevalent among sexual minorities compared to heterosexual populations (Hughes et al., 2016b), and while participants who reported alcohol use before or during sex had lower ED scores compared to older gay men who did not, the relationship was non-statistically significant. In the short term, alcohol use has stress-reducing effects (Pohorecky, 1981; Sillaber & Henniger, 2004) and may be the motive for older gay men to use before or during sex to focus on the sexual activity and not their sexual anxiety (Pyke, 2020). Although alcohol use before or during sex did not predict ED when other factors were controlled, future studies might examine this factor further in relation to ED among older gay men.

Internalized homophobia was included in the multivariate model, as it is a unique stressor and commonly experienced by gay men (Herek et al., 1998). However, no relationship was found between internalized homophobia and ED in this sample. Our null finding is opposite of a prior study that examined internalized homophobia and sexual dysfunction among lesbian, bisexual, and gay adults (Kuyper & Vanwesenbeeck, 2011). The different finding in our study may be explained by the focus on older gay men aged 50 and older. Additionally, participants in our study reported experiencing low levels of internalized homophobia, which may contribute to the nonsignificant association between internalized homophobia and ED.

In terms of race, participants who identified as other, multiracial, or multiethnic had lower ED compared to Black participants, which is interesting as a previous study found that Black men had higher prevalence of ED than White men (Selvin et al., 2007). However, race and ethnicity were no longer significant when physical activity and alcohol use before or during sex were controlled. Thus, racial differences may at least partially be explained by health behavior disparities such as physical activity level or alcohol use before or during sex, but future studies should continue to explore the role of intersectional identities and ED risk.

### Implications

These findings suggest the importance of overall health and sexual functioning as older gay men age. Clinicians and educators may find these results help them to better understand the contributing factors of ED among their older gay men clients or patients. A holistic assessment, such as a biopsychosocial assessment, may be vital to assess older gay men’s sexual health and determine possible interventions as the current study confirmed that sociodemographic characteristics are known to be associated with ED.

### Limitations

Although this study represents a novel exploration into factors associated with ED among older gay men in the Midwestern United States, the results should be viewed with caution. The sample was predominantly White, highly educated, and with high socioeconomic status, and was restricted to older gay men in the Midwest. Social acceptability and laws regarding LGBTQIA + issues vary from region to region and may have influenced how participants responded. Furthermore, the current study utilized a cross-sectional design, which does not allow for causal inferences to be made; future studies might use longitudinal designs to address this limitation. Additionally, health items were assessed using participant self-reporting for several items that assessed health, which increases risk of recall bias or misreporting. Finally, future studies may consider collecting a larger sample to reduce potential type II error.

### Conclusion

Age and overall health were found to be associated with ED among older gay men in the Midwestern United States. While this trend is similar to general populations of men, this study reinforces the importance of health as an integral piece of healthy sexual functioning among older gay men. Thus, clinicians and educators must highlight the importance of health-promoting behaviors, especially among older adult clients who present with sexual dysfunction. Additionally, it is essential to understand the potential for social and structural facets that influence health and how these may affect a multitude of avenues of gay men’s lives. Future studies should continue to investigate the factors associated with ED in older gay men and examine this phenomenon among a more diverse sample.

## Data Availability

The data that support the findings of this study are available from the corresponding author, Lucas R. Prieto, upon reasonable request.

## Code Availability

IBM SPSS V.28.

## References

[CR1] Aytaç, I. A., Araujo, A. B., Johannes, C. B., Kleinman, K. P., & McKinlay, J. B. (2000). Socioeconomic factors and incidence of erectile dysfunction: Findings of the longitudinal Massachussetts Male Aging Study. *Social Science & Medicine,**51*(5), 771–778. 10.1016/S0277-9536(00)00022-8 10975236 10.1016/S0277-9536(00)00022-8

[CR2] Bancroft, J., Carnes, L., Janssen, E., Goodrich, D., & Long, J. S. (2005). Erectile and ejaculatory problems in gay and heterosexual men. *Archives of Sexual Behavior,**34*(3), 285–297. 10.1007/s10508-005-3117-7 15971011 10.1007/s10508-005-3117-7

[CR3] Barbonetti, A., D’Andrea, S., Cavallo, F., Martorella, A., Francavilla, S., & Francavilla, F. (2019). Erectile dysfunction and premature ejaculation in homosexual and heterosexual men: A systematic review and meta-analysis of comparative studies. *Journal of Sexual Medicine,**16*(5), 624–632. 10.1016/j.jsxm.2019.02.014 30926517 10.1016/j.jsxm.2019.02.014

[CR4] Brown, H. (2011). Exercising choice: The economic determinants of physical activity behaviour of an employed population. *Social Science & Medicine,**73*(3), 383–390. 10.1016/j.socscimed.2011.06.001 21757272 10.1016/j.socscimed.2011.06.001 PMC3162476

[CR5] Burchardt, M., Burchardt, T., Baer, L., Kiss, A. J., Pawar, R. V., Shabsigh, A., De La Taille, A., Hayek, O. R., & Shabsigh, R. (2000). Hypertension is associated with severe erectile dysfunction. *Journal of Urology,**164*(4), 1188–1191. 10.1016/S0022-5347(05)67138-8 10992363 10.1016/S0022-5347(05)67138-8

[CR6] Centers for Disease Control and Prevention. (2011). *Behavioral risk factor surveillance system questionnaire*. U.S. Department of Health and Human Services. https://www.cdc.gov/brfss/questionnaires/pdf-ques/2011brfss.pdf

[CR7] Centers for Disease Control and Prevention. (2019). *2019 BRFSS Survey data and documentation*. U.S. Department of Health and Human Services. https://www.cdc.gov/brfss/annual_data/annual_2019.html

[CR8] Davison, S. L., Bell, R. J., LaChina, M., Holden, S. L., & Davis, S. R. (2009). The relationship between self-reported sexual satisfaction and general well-being in women. *Journal of Sexual Medicine,**6*(10), 2690–2697. 10.1111/j.1743-6109.2009.01406.x 19817981 10.1111/j.1743-6109.2009.01406.x

[CR9] Dogan, T., Tugut, N., & Golbasi, Z. (2013). The relationship between sexual quality of life, happiness, and satisfaction with life in married Turkish women. *Sexuality and Disability,**31*(3), 239–247. 10.1007/s11195-013-9302-z 10.1007/s11195-013-9302-z

[CR10] Emlet, C. A., Fredriksen-Goldsen, K. I., Kim, H.-J., & Jung, H. (2020). Accounting for HIV health disparities: Risk and protective factors among older gay and bisexual men. *Journal of Aging and Health,**32*(7–8), 677–687. 10.1177/0898264319848570 31079525 10.1177/0898264319848570 PMC6851439

[CR11] Feldman, H. A., Goldstein, I., Hatzichristou, D. G., Krane, R. J., & McKinlay, J. B. (1994). Impotence and its medical and psychosocial correlates: Results of the Massachusetts Male Aging Study. *Journal of Urology,**151*(1), 54–61. 10.1016/S0022-5347(17)34871-1 8254833 10.1016/S0022-5347(17)34871-1

[CR12] Filice, E., Raffoul, A., Meyer, S. B., & Neiterman, E. (2019). The influence of Grindr, a geosocial networking application, on body image in gay, bisexual and other men who have sex with men: An exploratory study. *Body Image,**31*, 59–70. 10.1016/j.bodyim.2019.08.007 31446375 10.1016/j.bodyim.2019.08.007

[CR13] Flesia, L., Monaro, M., Jannini, E. A., & Limoncin, E. (2023). “I’m too old for that”: The role of ageism and sexual dysfunctional beliefs in sexual health in a sample of heterosexual and LGB older adults: A pilot study. *Healthcare,**11*(4), 459. 10.3390/healthcare11040459 36832993 10.3390/healthcare11040459 PMC9957165

[CR14] Foy, C. G., Newman, J. C., Berlowitz, D. R., Russell, L. P., Kimmel, P. L., Wadley, V. G., Thomas, H. N., Lerner, A. J., & Riley, W. T. (2019). Blood pressure, sexual activity, and erectile function in hypertensive men: Baseline findings from the Systolic Blood Pressure Intervention Trial (SPRINT). *Journal of Sexual Medicine,**16*(2), 235–247. 10.1016/j.jsxm.2018.12.007 30655182 10.1016/j.jsxm.2018.12.007 PMC6444897

[CR15] Gandaglia, G., Briganti, A., Jackson, G., Kloner, R. A., Montorsi, F., Montorsi, P., & Vlachopoulos, C. (2014). A systematic review of the association between erectile dysfunction and cardiovascular disease. *European Urology,**65*(5), 968–978. 10.1016/j.eururo.2013.08.023 24011423 10.1016/j.eururo.2013.08.023

[CR17] Gitlitz, T., & Ayalon, L. (2023). The mediating role of self-directed ageism in sexual health among Jewish-Israeli older people. *Geriatric Nursing,**54*, 341–349. 37952297 10.1016/j.gerinurse.2023.10.006

[CR18] Gonzales, G., & Henning-Smith, C. (2017). Health disparities by sexual orientation: Results and implications from the Behavioral Risk Factor Surveillance System. *Journal of Community Health,**42*, 1163–1172. 28466199 10.1007/s10900-017-0366-z

[CR19] Harrell, F. E. (2001). *Regression modeling strategies: With applications to linear models, logistic regression, and survival analysis*. Springer.

[CR20] Heikkilä, A., Kaipia, A., Venermo, M., Kautiainen, H., & Korhonen, P. (2017). Relationship of blood pressure and erectile dysfunction in men without previously diagnosed hypertension. *Journal of Sexual Medicine,**14*(11), 1336–1341. 10.1016/j.jsxm.2017.09.007 28993149 10.1016/j.jsxm.2017.09.007

[CR21] Herek, G. M., Gillis, J. R., & Cogan, J. C. (2009). *Revised internalized homophobia scale (IHP-R)* [Database record]. APA PsycTests. 10.1037/t10966-000

[CR22] Herek, G. M., Cogan, J. C., Gillis, J. R., & Glunt, E. K. (1998). Correlates of internalized homophobia in a community sample of lesbians and gay men. *Journal of the Lesbian Medical Association,**2*, 17–26.

[CR23] Holmberg, D., Blair, K. L., & Phillips, M. (2010). Women’s sexual satisfaction as a predictor of well-being in same-sex versus mixed-sex relationships. *Journal of Sex Research,**47*(1), 1–11. 10.1080/00224490902898710 19381998 10.1080/00224490902898710

[CR24] Hughes, J. L., Camden, A. A., & Yangchen, T. (2016a). Rethinking and updating demographic questions: Guidance to improve descriptions of research samples. *Psi Chi Journal of Psychological Research,**21*(3), 138–151. 10.24839/2164-8204.JN21.3.138 10.24839/2164-8204.JN21.3.138

[CR25] Hughes, T. L., Wilsnack, S. C., & Kantor, L. W. (2016b). The influence of gender and sexual orientation on alcohol use and alcohol-related problems: Toward a global perspective. *Alcohol Research: Current Reviews,**38*(1), 121–132. 27159819 10.35946/arcr.v38.1.15 PMC4872607

[CR26] IBM SPSS Statistics for Windows. (2021). *version 28*. IBM Corp.

[CR27] Jackson, C. L., Agénor, M., Johnson, D. A., Austin, S. B., & Kawachi, I. (2016). Sexual orientation identity disparities in health behaviors, outcomes, and services use among men and women in the United States: A cross-sectional study. *BMC Public Health,**16*. 10.1186/s12889-016-3467-1 10.1186/s12889-016-3467-1 PMC4989521 27534616

[CR28] Kattari, S. K., Kattari, L., Johnson, I., Lacombe-Duncan, A., & Misiolek, B. A. (2020). Differential experiences of mental health among trans/gender diverse adults in Michigan. *International Journal of Environmental Research and Public Health,**17*(18), 6805. 10.3390/ijerph17186805 32961959 10.3390/ijerph17186805 PMC7557385

[CR29] Knyazev, G. G., Slobodskaya, H. R., Kharchenko, I. I., & Wilson, G. D. (2004). *Substance use measures* [Database record]. APA PsycTests. 10.1037/t12312-000

[CR30] Kroenke, K., Spitzer, R. L., & Williams, J. B. (2003). The Patient Health Questionnaire-2: Validity of a two-item depression screener. *Medical Care,**41*(11), 1284–1292. 10.1097/01.MLR.0000093487.78664.3C 14583691 10.1097/01.MLR.0000093487.78664.3C

[CR31] Kuyper, L., & Vanwesenbeeck, I. (2011). Examining sexual health differences between lesbian, gay, bisexual, and heterosexual adults: The role of sociodemographics, sexual behavior characteristics, and minority stress. *Journal of Sex Research,**48*(2–3), 263–274. 10.1080/00224491003654473 20191420 10.1080/00224491003654473

[CR32] Latini, D. M., Penson, D. F., Wallace, K. L., Lubeck, D. P., & Lue, T. F. (2006). Erectile dysfunction: Clinical and psychosocial characteristics of men with erectile dysfunction: Baseline data from ExCEED. *Journal of Sexual Medicine,**3*(6), 1059–1067. 10.1111/j.1743-6109.2006.00331.x 17100939 10.1111/j.1743-6109.2006.00331.x

[CR33] Laumann, E. O., West, S., Glasser, D., Carson, C., Rosen, R., & Kang, J.-H. (2007). Prevalence and correlates of erectile dysfunction by race and ethnicity among men aged 40 or older in the United States: From the Male Attitudes Regarding Sexual Health Survey. *Journal of Sexual Medicine,**4*(1), 57–65. 10.1111/j.1743-6109.2006.00340.x 17081223 10.1111/j.1743-6109.2006.00340.x

[CR34] Levy, B. (2009). Stereotype embodiment: A psychosocial approach to aging. *Current Directions in Psychological Science,**18*(6), 332–336. 10.1111/j.1467-8721.2009.01662.x 20802838 10.1111/j.1467-8721.2009.01662.x PMC2927354

[CR35] Lindau, S. T., Schumm, L. P., Laumann, E. O., Levinson, W., O’Muircheartaigh, C. A., & Waite, L. J. (2007). A study of sexuality and health among older adults in the United States. *New England Journal of Medicine,**357*(8), 762–774. 10.1056/NEJMoa067423 17715410 10.1056/NEJMoa067423 PMC2426743

[CR36] Liu, Q., Zhang, Y., Wang, J., Li, S., Cheng, Y., Guo, J., Tang, Y., Zeng, H., & Zhu, Z. (2018). Erectile dysfunction and depression: A systematic review and meta-analysis. *Journal of Sexual Medicine,**15*(8), 1073–1082. 10.1016/j.jsxm.2018.05.016 29960891 10.1016/j.jsxm.2018.05.016

[CR37] Mannino, D. M., Klevens, R. M., & Flanders, W. D. (1994). Cigarette smoking: An independent risk factor for impotence? *American Journal of Epidemiology,**140*(11), 1003–1008. 10.1016/s0022-5347(17)38444-6 7985647 10.1016/s0022-5347(17)38444-6

[CR38] McDonagh, L. K., Stewart, I., Morrison, M. A., & Morrison, T. G. (2016). Development and psychometric evaluation of the Gay Male Sexual Difficulties Scale. *Archives of Sexual Behavior,**45*(6), 1299–1315. 10.1007/s10508-015-0664-4 26728054 10.1007/s10508-015-0664-4

[CR39] Meyer, I. H. (1995). Minority stress and mental health in gay men. *Journal of Health and Social Behavior,**36*, 38–56. 7738327 10.2307/2137286

[CR40] Morgentaler, A. (1999). Male impotence. *The Lancet,**354*(9191), 1713–1718. 10.1016/S0140-6736(99)06586-1 10.1016/S0140-6736(99)06586-1 10568586

[CR41] Mulhall, J. P., Luo, X., Zou, K. H., Stecher, V., & Galaznik, A. (2016). Relationship between age and erectile dysfunction diagnosis or treatment using real-world observational data in the USA. *International Journal of Clinical Practice,**70*(12), 1012–1018. 10.1111/ijcp.12908 28032424 10.1111/ijcp.12908 PMC5540144

[CR42] National Health and Nutrition Examination Survey. (2007). *2005–2006 data documentation, codebook, and frequencies*. https://wwwn.cdc.gov/Nchs/Nhanes/2005-2006/DIQ_D.htm

[CR43] Nelson, C. J., Mulhall, J. P., & Roth, A. J. (2011). The association between erectile dysfunction and depressive symptoms in men treated for prostate cancer. *Journal of Sexual Medicine,**8*(2), 560–566. 10.1111/j.1743-6109.2010.02127.x 21155979 10.1111/j.1743-6109.2010.02127.x

[CR44] Operario, D., Gamarel, K. E., Grin, B. M., Lee, J. H., Kahler, C. W., Marshall, B. D., van den Berb, J. J., & Zaller, N. D. (2015). Sexual minority health disparities in adult men and women in the United States: National Health and Nutrition Examination Survey, 2001–2010. *American Journal of Public Health,**105*(10), e27–e34. 10.2105/AJPH.2015.302762 26270288 10.2105/AJPH.2015.302762 PMC4566530

[CR45] Parsons, J. T., Starks, T. J., DuBois, S., Grov, C., & Golub, S. A. (2013). Alternatives to monogamy among gay male couples in a community survey: Implications for mental health and sexual risk. *Archives of Sexual Behavior,**42*(2), 303–312. 10.1007/s10508-011-9885-3 22187028 10.1007/s10508-011-9885-3 PMC5830303

[CR46] Penson, D. F., Wessells, H., Cleary, P., & Rutledge, B. N. (2009). Sexual dysfunction and symptom impact in men with long-standing type 1 diabetes in the DCCT/EDIC cohort. *Journal of Sexual Medicine,**6*(7), 1969–1978. 10.1111/j.1743-6109.2009.01292.x 19453899 10.1111/j.1743-6109.2009.01292.x PMC2861494

[CR47] Pizzol, D., Demurtas, J., Stubbs, B., Soysal, P., Mason, C., Isik, A. T., Solmi, M., Smith, L., & Veronese, N. (2019). Relationship between cannabis use and erectile dysfunction: A systematic review and meta-analysis. *American Journal of Men’s Health,**13*(6), 1557988319892464. 10.1177/1557988319892464 31795801 10.1177/1557988319892464 PMC6893937

[CR48] Pohorecky, L. (1981). The interaction of alcohol and stress: A review. *Neuroscience & Biobehavioral Reviews,**5*(2), 209–229. 10.1016/0149-7634(81)90003-8 6115346 10.1016/0149-7634(81)90003-8

[CR49] Pyke, R. E. (2020). Sexual performance anxiety. *Sexual Medicine Reviews,**8*(2), 183–190. 10.1016/j.sxmr.2019.07.001 31447414 10.1016/j.sxmr.2019.07.001

[CR50] Rhodes, S. D., McCoy, T., Hergenrather, K. C., Omli, M. R., & DuRant, R. H. (2007). Exploring the health behavior disparities of gay men in the United States: Comparing gay male university students to their heterosexual peers. *Journal of LGBT Health Research,**3*(1), 15–23. 10.1300/J463v03n01_03 18029312 10.1300/J463v03n01_03

[CR51] Rosen, R. C., & Marin, H. (2003). Prevalence of antidepressant-associated erectile dysfunction. *Journal of Clinical Psychiatry,**64*, 5–10. 12971810

[CR52] Saigal, C. S., Wessells, H., Pace, J., Schonlau, M., Wilt, T. J., & Urologic Diseases in America Project. (2006). Predictors and prevalence of erectile dysfunction in a racially diverse population. *Archives of Internal Medicine,**166*(2), 207–212. 10.1001/archinte.166.2.207 16432090 10.1001/archinte.166.2.207

[CR54] Selvin, E., Burnett, A. L., & Platz, E. A. (2007). Prevalence and risk factors for erectile dysfunction in the US. *American Journal of Medicine,**120*(2), 151–157. 10.1016/j.amjmed.2006.06.010 17275456 10.1016/j.amjmed.2006.06.010

[CR55] Shabsigh, R., Klein, L. T., Seidman, S., Kaplan, S. A., Lehrhoff, B. J., & Ritter, J. S. (1998). Increased incidence of depressive symptoms in men with erectile dysfunction. *Urology,**52*(5), 848–852. 10.1016/S0090-4295(98)00292-1 9801112 10.1016/S0090-4295(98)00292-1

[CR56] Shiri, R., Koskimäki, J., Tammela, T. L., Häkkinen, J., Auvinen, A., & Hakama, M. (2007). Bidirectional relationship between depression and erectile dysfunction. *Journal of Urology,**177*(2), 669–673. 10.1016/j.juro.2006.09.030 17222655 10.1016/j.juro.2006.09.030

[CR57] Sillaber, I., & Henniger, M. S. (2004). Stress and alcohol drinking. *Annals of Medicine,**36*(8), 596–605. 10.1080/07853890410018862 15768831 10.1080/07853890410018862

[CR58] Silva, A. B., Sousa, N., Azevedo, L. F., & Martins, C. (2017). Physical activity and exercise for erectile dysfunction: Systematic review and meta-analysis. *British Journal of Sports Medicine,**51*(19), 1419–1424. 10.1136/bjsports-2016-096418 27707739 10.1136/bjsports-2016-096418

[CR59] Slevin, K. F., & Linneman, T. J. (2010). Old gay men’s bodies and masculinities. *Men and Masculinities,**12*(4), 483–507. 10.1177/1097184X08325225 10.1177/1097184X08325225

[CR60] Smith, J. F., Caan, B. J., Sternfeld, B., Haque, R., Quesenberry, C. P., Quinn, V. P., & Van Den Eeden, S. K. (2009). Racial disparities in erectile dysfunction among participants in the California Men’s Health Study. *Journal of Sexual Medicine,**6*(12), 3433–3439. 10.1111/j.1743-6109.2009.01519.x 19796017 10.1111/j.1743-6109.2009.01519.x

[CR61] Smith, L. J., Mulhall, J. P., Deveci, S., Monaghan, N., & Reid, M. (2007). Sex after seventy: A pilot study of sexual function in older persons. *Journal of Sexual Medicine,**4*(5), 1247–1253. 10.1111/j.1743-6109.2007.00568.x 17727349 10.1111/j.1743-6109.2007.00568.x

[CR62] Sprecher, S., & Cate, R. M. (2004). Sexual satisfaction and sexual expression as predictors of relationship satisfaction and stability. In J. H. Harvey, A. Wenzel, & S. Sprecher (Eds.), *The handbook of sexuality in close relationships* (pp. 235–256). Lawrence Erlbaum Associates.

[CR63] Syme, M. L., & Cohn, T. J. (2021). Aging sexual stereotypes and sexual expression in mid- and later life: Examining the stereotype matching effect. *Aging & Mental Health,**25*(8), 1507–1514. 10.1080/13607863.2020.1758909 32363907 10.1080/13607863.2020.1758909

[CR64] Tomlinson, J., & Wright, D. (2004). Impact of erectile dysfunction and its subsequent treatment with sildenafil: Qualitative study. *British Medical Journal,**328*(7447), 1037. 10.1136/bmj.38044.662176.EE 15051618 10.1136/bmj.38044.662176.EE PMC403839

[CR65] Tostes, R. C., Carneiro, F. S., Lee, A. J., Giachini, F. R. C., Leite, R., Osawa, Y., & Webb, R. C. (2008). Cigarette smoking and erectile dysfunction: Focus on NO bioavailability and ROS generation. *Journal of Sexual Medicine,**5*(6), 1284–1295. 10.1111/j.1743-6109.2008.00804.x 18331273 10.1111/j.1743-6109.2008.00804.x PMC3003263

[CR66] Turner, R. J., Thomas, C. S., & Brown, T. H. (2016). *Self-rated health measure* [Database record]. APA PsycTests. 10.1037/t58992-000

[CR67] Wight, R. G., LeBlanc, A. J., Meyer, I. H., & Harig, F. A. (2015). Internalized gay ageism, mattering, and depressive symptoms among midlife and older gay-identified men. *Social Science & Medicine,**147*, 200–208. 10.1016/j.socscimed.2015.10.066 26588435 10.1016/j.socscimed.2015.10.066 PMC4689679

[CR68] Williams, D. R., & Cooper, L. A. (2019). Reducing racial inequities in health: Using what we already know to take action. *International Journal of Environmental Research and Public Health,**16*(4), 606. 10.3390/ijerph16040606 30791452 10.3390/ijerph16040606 PMC6406315

